# Effect of microthread design on the preservation of marginal bone around immediately placed implants: a 5-years prospective cohort study

**DOI:** 10.1186/s12903-021-01881-w

**Published:** 2021-10-21

**Authors:** Hoori Aslroosta, Solmaz Akbari, Nima Naddafpour, Seyed Taha Adnaninia, Afshin Khorsand, Niusha Namadmalian Esfahani

**Affiliations:** 1 Periodontics Department, Dental Faculty, Tehran University of Medical Sciences, Tehran, Iran; 2 Periodontics Department, Dental Implant Research Center, Dentistry Research Institute, Dental Faculty, Tehran University of Medical Sciences, Tehran, Iran; 3 Periodontics Department, Dental Faculty, Islamic Azad University, Tehran, Iran; 4 General Dentist, Tehran, Iran; 5 Periodontics Department, Dental Faculty, Semnan University of Medical Sciences, Semnan, Iran

**Keywords:** Implant designs, Immediate implant, Microthread, Marginal bone loss

## Abstract

**Background:**

This study aimed to evaluate the effect of the microthread design at the implant neck on the preservation of marginal bone around immediately-placed implants in a 5-year follow up.

**Methods:**

Thirty patients received 41 immediately placed implants which were randomly assigned to treatment groups with microthreaded implants (test group, n = 22) or threaded implants (control group, n = 19). Clinical and radiographic analyses were carried out after 1 and5 years. Plaque index, bleeding on probing, suppuration, probing depth and marginal bone loss were subject to evaluations. The results were analyzed with the T-test, Fisher’s exact test and Mann–Whitney U test.

**Results:**

No implants failed; thirty-five implants (in 27 patients); 21 microthreaded and 14 threaded implants; completed the 5 year follow up. The mean values of the marginal bone loss in microthreaded and threaded groups were 1.12 ± 0.95 mm and 0.87 ± 0.78 mm, respectively during an observation period of 70.9 ± 10.4 months; the differences in marginal bone loss and other pre-implant parameters were not significant between groups (*P* > 0.05).

**Conclusion:**

Both implant designs showed acceptable results in terms of the clinical parameters and marginal bone level. Within the limitation of this study, the results did not demonstrate any superiority of the microthread design compared to threaded one in marginal bone preservation around immediately placed implants over 5 years of loading.

## Background

The gradual peri-implant crestal bone remodeling will occur once implants have been placed [1]. It is a complex multi-factorial phenomenon and is influenced by several clinician-, patient-, and implant-related factors [2, 3]. Scientific endeavors have been recently made to preserve the crestal bone as coronally as possible to improve long term success and to obtain optimum aesthetic results of implant therapy [4, 5]. Accordingly, several macro- and micro-design features of implants have been introduced to improve marginal bone maintenance [6–10]. One of these modifications is addition of microthreads in the coronal part of implants [8, 11–16]. As shown in finite element analysis studies, the microthraed design allows a better distribution of stress to the surrounding bone and may minimize the marginal bone loss (MBL) [17–20]. Several clinical studies have also demonstrated that rough surfaced implants with microthreads at the neck can better resist axial loads and preserve the marginal bone level during the healing period; therefore, resulting in less MBL under functional loading [6, 7, 11, 21–23] But microthreads designed on implant neck surfaces have been reported to provide controversial clinical outcomes [12, 24]. In this regard, in a recent systematic review, no significant differences were found between microthreaded and conventionally rough neck implants in terms of marginal bone loss [25]. It should be noted that majority of these aforementioned studies have reported short term results and compared microthreaded-neck implants with implants with a non-retentive configuration in the neck area, including machined or conventional rough surface.

There has been insufficient evidence whether enhancement of implant surface via addition of microthreads on implant necks could affect marginal bone loss in long term. Our first randomized clinical trial study revealed comparable levels of bone loss after 6 and 12 months in microthreaded and threaded implants placed immediately in post-extraction sockets [26]. The rationale behind this study was to carry out an evaluation of the long-term marginal bone level in microthreaded implants in comparison to macroscopically similar implants without microthreads, placed immediately in post-extraction sockets.

## Methods

This study describes a 5-year follow-up of the cases previously included in a parallel randomized clinical trial study which evaluated the effects of a microthread design on the marginal bone level around immediately placed implants in extraction sockets [26]. The study protocol was approved by the Ethics Committee of Tehran University of Medical Sciences (IR.TUMS.REC.1394.686). The patients were completely informed of being included in the study and all signed the informed consent forms accordingly.

### Study protocol

**Fig. 1:**
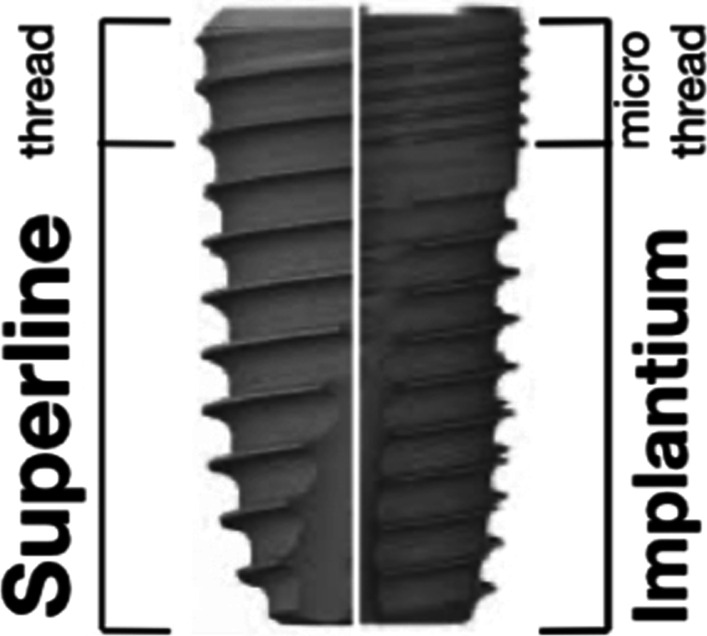
The evaluated implants. In the left, the implant without microthread as the control group (Superline®). In the right, the implant with microthread in the coronal part as the test group (Implantium®)

Study protocol were described in detail previously [26]. In brief, study subjects were consecutively recruited among patients referred to the implant department of Tehran university of medical sciences, Tehran, Iran who in need of a tooth extraction and immediate implant placement in anterior segment of maxilla (second premolar to second premolar). Inclusion criteria applied were as follow: at least one hopeless tooth due to endodontic or prosthetic reasons, presence of at least 2 mm height and 1 mm thickness of keratinized mucosa, good oral hygiene, intact facial socket wall after tooth extraction and presence of at least 3 mm bone beyond the apex to achieve acceptable primary stability. Exclusion criteria included any signs of parafunctional habits, uncontrolled diabetes mellitus, cigarette smoking (greater than 10 cigarettes per day), pregnant or lactating, active periodontal diseases and periapical radiolucency in the radiographic view. After atraumatic tooth extraction, the test group received implants with microthreads on the coronal portion of the fixture (Implantium®, Dentium, Seoul, South Korea), while the control group was made up of those with microthread-free implants (Superline®, Dentium, Seoul, South Korea). In their crest module, both implants had a sandblast large grit acid etch surface and 0.5-mm of a tapered bevel machine surface; there was a conical hex connection between the implant and abutment. Except for the coronal 2 mm of the fixture, they were identical in their geometry (Fig. 1). All implants were submerged 1–2 mm apical to the facial bony wall of extraction sockets. All cases were devoid of any substitute bone graft materials even in the buccal gap. Cover screwed were secured and sutures were removed 10 days later. All the implants received cemented prostheses (Auritex-40; Aurident Inc., Fullerton, CA) with a 4–5 mm occlusal platform during a 3 to 4-months period. Patients were met at baseline, 1 and 5 years after operation for clinical and radiographic examinations. The first study had been conducted on 30 patients with 41 implants placed in fresh extraction sites of the anterior segment of maxilla. After a mean follow-up of 70.9 ± 10.4 months, 27 patients (out of 30) with 35 implants have completed the follow-up period. Drop out reasons varied: 2 implants in 1 patient were excluded from the study due to severe bone loss (failing implants). One patient with 3 implants had already changed the place of residence and was out of reach. One patient with one implant refused to return for the follow-up visits.

### Clinical examination

During the 5-year follow-up, the following clinical parameters were measured and recorded among which MBL defined as a primary outcome and PI, Probing depth, BOP, mucosal recession and absence of keratinized mucosa were considered as a secondary outcome.

- Plaque index (PI): presence of plaque (yes) and the absence of plaque (no).
- Probing depth was measured at four points of each implant: mesial, distal, mid-buccal and mid-lingual and reported as mean value.
- Bleeding on probing (BOP): ‘yes/ no’. BOP was reported as a number and also percentage of implants which had bleeding [27].
- Mucosal recession: ‘yes’ providing either the margin of prosthesis or the body of implant was visible on the buccal aspect.
- Keratinized mucosa: ‘presence’ (in case of the attached mucosa of over 0.5 mm)/absence [27].

### Radiographic examination

**Fig. 2:**
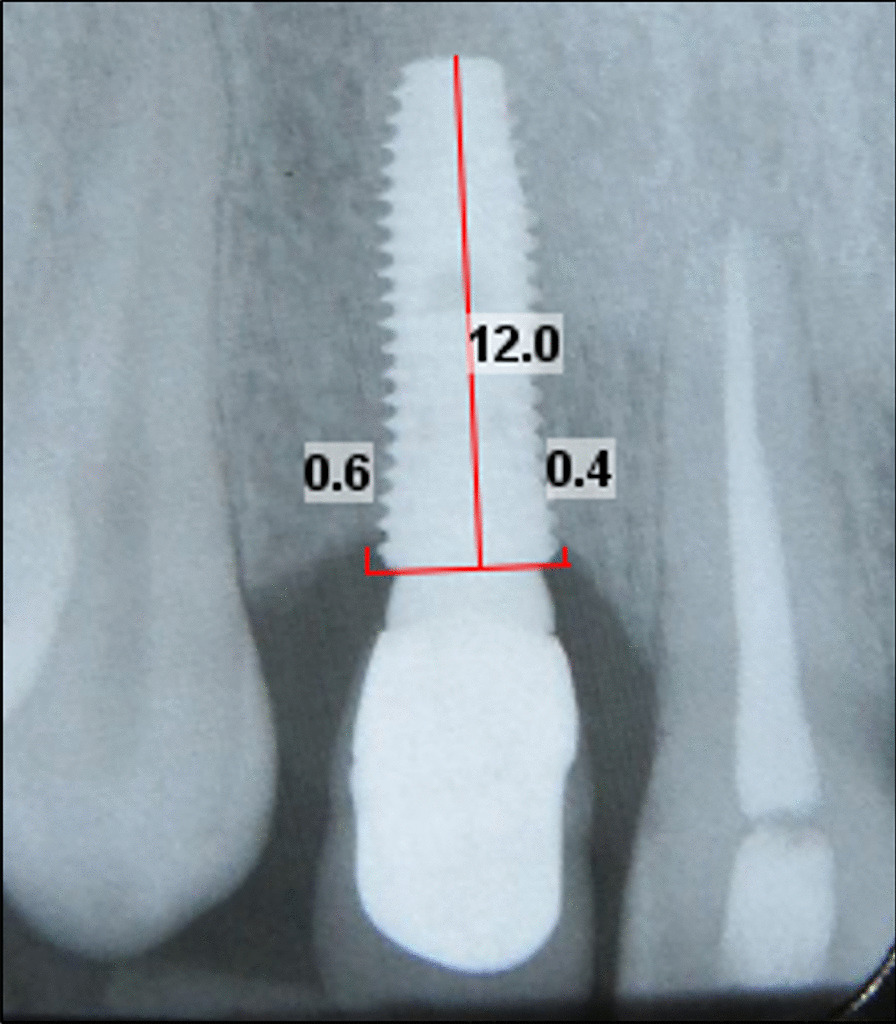
After calibration of radiographs by the length of each implant, marginal bone loss measured as vertical distance between implant shoulder and the marginal bone

Parallel periapical radiographs were obtained with the long cone technique using XCP (XCP instruments; Rinn Corporation Elgin, Elgin, IL, USA). Radiographs were imported to software Romexis® (Planmeca, IL, USA) version 2.3.1.R. X-ray calibration was performed in line with the length of any single implant. One calibrated examiner (S.A) measured the distance between the implant shoulder and marginal bone (by mm) at mesial and distal aspect of each implant and recorded it as the marginal bone loss (MBL). To assess the inter examiner variability, 5 radiographs (15% of samples) were randomly selected (based on a table of random numbers) to repeat MBL measurement. Then the clinical and radiographic findings were compared with those at the baseline (Fig. 2).

### Statistical analysis

Blinded to the data, an independent statistician was requested to review the methodology of the study and perform the statistical analysis as well. A Mann–Whitney U test was administered to compare the mean probing depths and bone loss at corresponding levels between the test and control groups. The total mean probing depth and the total bone loss were compared by using T-test between study groups. A Fisher’s exact test was utilized to compare the results of clinical examinations including BOP, plaque index, keratinized mucosa and mucosal recession between the two groups. The SPSS software (IBM Corp. Released 2011. IBM SPSS Statistics for Windows, Version 20.0. Armonk, NY: IBM Corp) was used for statistical analyses. *P* < 0.05 was set the significant level.

## Results

**Fig. 3:**
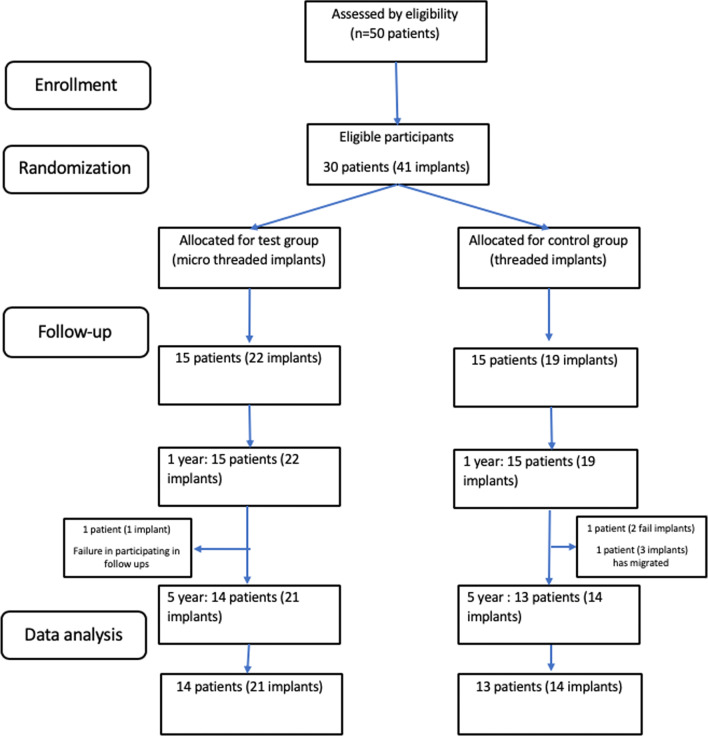
CONSORT Flow chart of the patients during study

The study evaluated 35 implants (out of all 41). This consisted of 21 implants (14 patients, 49.7 ± 10.8 years) in the microthreaded (test) and 14 implants (13 patients, 43.6 ± 11.6 years) in the threaded (control) group (Fig. 3). All of these implants became successfully osseointegrated and were in function.

Table 1 illustrates all the clinical conditions of implants in both groups. The Fisher’s exact test did not show any significant differences in plaque index, bleeding on probing, the presence of suppuration, the absence of attached gingiva and the mucosal recession between the two groups (*P* > 0.05). The mean probing depths in the test and control groups were 2.58 ± 1.28 and 1.90 ± 0.55 mm, respectively. The T-test did not show any significant difference between groups (*P* > 0.05) (Table 1).

**Table 1:** The peri-implant clinical parameters of the implants in the two groups

	Test group	Control group	*p* value
PI count (%)	14 (66.7)	7 (53.84)	0.582
BOP count (%)	16 (76.2)	6 (46.15)	0.196
SUP count (%)	0	0	–
Mean probing depth (mean ± SD) (mm)	2.58 ± 1.28	1.90 ± 0.55	0.108
Mucosal recession count (%)	6 (28.6)	2 (15.38)	0.425
Absence of Keratinized mucosa count (%)	5 (23.8)	3 (23.07)	0.575

Significance level: *P* < 0.05

**Fig. 4:**
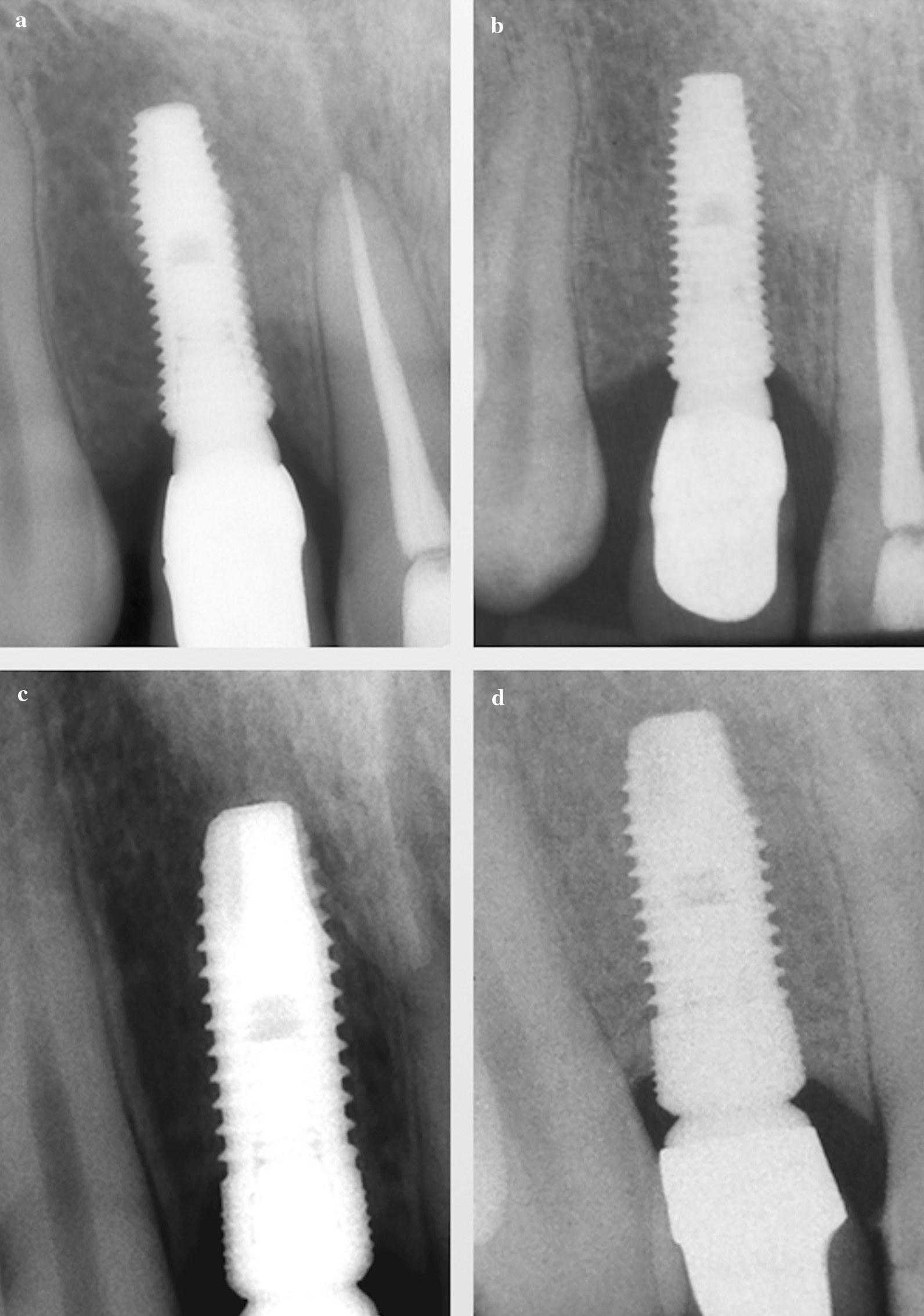
Intraoral periapical radiographs in treaded implant (**a** 1-year and **b** 5-year visit); and microthreaded implant (**c** 1-year and **d** 5-year visit)

One year after the loading, the mean marginal bone loss in the test and control groups was 0.75 ± 0.32 and 0.71 ± 0.41 mm respectively; the figures experienced a rise to 1.12 ± 0.95 and 0.87 ± 0.78 mm after mean 70.9 ± 10.4 months of function (Fig. 4). Although the mean MBL in control group was less than that of the test group, the difference did not touch the significant level (*P* > 0.05) (Table 2).

**Table 2:** MBL in test and control group after 1 and 5 years of loading

Time point	Test group Mean ± SD (mm)	Control group Mean ± SD (mm)	*P* value
1 year	0.75 ± 0.32	0.71 ± 0.41	0.21
5 year	1.12 ± 0.95	0.87 ± 0.78	0.461

Significance level: *P* < 0.05

## Discussion

In this prospective study, the amount of MBL was evaluated in a 5-year period around either microthreaded or threaded implants that had been placed immediately after tooth extraction. After mean 70.9 ± 10.4 months of function, the mean MBL ranged from 0.87 to 1.12 mm. The crestal bone loss in both groups was within the limits of implant success criterion. [28, 29]

Parallel to our results, in a long-term prospective study on immediately placed implants, Covani et al. [30] observed that 82% of implants experienced MBL of 0.6 to 1.5 mm at a 10-year follow-up. Other systematic reviews showed that immediately placed implants had an acceptable marginal bone stability similar to implants placed in a healed bone [31, 32].

In the present study, the mean MBL in the test and control groups saw an increase from 0.75 ± 0.32 mm in a 1 year follow-up to 1.12 ± 0.95 mm after 5 years and from 0.71 ± 0.41 mm to 0.87 ± 0.78 mm respectively. Although long-term MBL in control group was less than test group at 5-year follow-up, the difference in the mean MBL between the two groups was not significant either in 1or 5-year visits. Thus, it seems that presence or absence of microthread may have no positive effect on long-term marginal bone preservation.

In stark contrast, Bratu et al. [22] (1-year follow up), Lee et al. [7] (3-year follow-up), and Nickening et al. [23] (5-year follow up) who found less crestal bone loss in microthreaded design. What may merit attention though is that the last three studies compared rough microthreaded implants with polished [22], rough thread-free [7] or machined [23] neck implants. In another study, Song et al. [11] used two types of implants with identical designs other than the location of micrethreads so that they could evaluate the impact of the microthread location on peri-implant bone level. The implants with microthreads up to the implant top showed less MBL compared to the other group in which microthreads started 0.5 mm below the implant top after 1 year of loading. The first design was identical to our test group implants, but in the other group there were no retentive elements on the coronal 0.5 mm of implant neck area, shown to be biomechanically effective in providing mechanical stimulus to preserve marginal bone [33].

Numerous studies have confirmed that a myriad of factors could exert a potential impact on peri-implant bone level. This may include the type of implant-abutment connections (platform switch/ matching abutment) [34, 35], geometric designs of connection types [36, 37], implant neck configurations [8, 25, 35], abutment heights and implant macro/microdesigns [35, 38–40]. What put an obstacle on the way of the authors to compare the results of studies was that the test and control implants did not solely differ in one aspect (the neck area) and were consequently subjected to the presence of confounding variables.

Therefore, in the present study the authors confined themselves to evaluating the differences in the neck design in order to discern the precise impact the microthreads had on MBL. Thus, they endeavored to maintain the overall geometrical designs as resembling as possible. These could be listed as the diameter and length of the implants, forms of the crest module and implant abutment connections (platform switching pattern), and surface texture were similar.

Conversely, in agreement with 1 and 5-year findings of this study, two RCTs by Kang et al. [12] and Spies et al. [24] used macro and micro-neck thread implants with platform switching concepts as the test and control groups. They similarly found no significant marginal bone loss between studied groups after 1 year of loading.

It has been suggested by finite element analysis (FEA), that the microthreaded design could lead to more compressive and less shear stress under off-axis loading and is less likely to pose a risk of MBL triggered by overloading [20]. However, the authors decided not to refer to such results for two reasons. First, FEA results should be generally interpreted and extrapolated to clinical states with caution [41] Furthermore, these studies applied 3-D FEA models to compare stress distribution of smooth and microthreaded implants installed in posterior of mandible or maxilla, while the present study relied on a clinical approach to evaluate macro- and micro-thread configurations in long term [17, 19].

Several clinical and animal studies have investigated the effectiveness of the microthread design on marginal bone preservation around implants placed in native bones, which are mostly in posterior sites and receive short term follow ups. Within the limits of our knowledge, the present study has been the only long-term assessment (after a mean 5 years of loading) of the effect microthread configurations might have on MBL around immediately placed dental implants. The results were void of any superiority of this design in terms of marginal bone preservation. This is in corroboration with an animal study by De Sancits et al., who placed a microthreaded implant and 3 other macro design implants in fresh extraction sockets in dogs [42]. They did not report any significant differences in implant-bone contact percentages, bone loss in the buccal area, bone healing patterns 6 weeks after the placement of implants.

Other considerable sources of difference in studies are the variations in radiographic imaging systems, reference points, and base line views in radiographic MBL evaluation. The higher level of bone resorption recorded in the present study could be attributed to different baseline views. Some studies took the baseline radiographic view after prosthesis delivery [11, 12, 43], and subsequently they did not calculate the highest amount of bone resorption, occurring between the time of implant placement and final prosthesis placement [44–46].

In more than 45% of implants in this study, PI and BOP indices were positive; this calls for further particular emphasis to be placed on plaque control measures and maintenance programs. A recent systematic review has depicted that immediate implantation could be associated with implant-based mucositis [47]. Deep sub gingival position of implant shoulder (especially in implants placed immediately) in the esthetic zone might predispose such implants to mucosal recession as a result of which more marginal bone loss happens [48].

The present study suffered from three main drawbacks. First, akin to the majority of studies in implant dentistry, the study relied on intraoral periapical radiographs in order to evaluate the marginal bone alterations. However, peri-apical radiographs are incapable to detect 3D configuration of periimplant bone level [49]; also, overestimate the MBL and it is influenced by many factors. Despite the fact that cone-beam CT radiographs enhance the precision of the information concerning buccal bone configurations (thickness and position) with a great impact on the long-term outcomes of implants in esthetic zone [50, 51], the authors decided not to take CBCT records for the growing concern of radiation dose and ethical constraints pertaining to healthy subjects.

Second, the latest studies have repeatedly underscored the facial wall thickness and the dimension of buccal gap between implant body and socket wall as important factors influencing the peri-implant bone position in immediately placed implants. At the time of the initial design, theses variables had not been noticed. Consequently, it is recommended to conduct further RCTs with a larger sample size considering these factors so that the study groups can be unified and the possible effects of the microthread design can be determined with a higher accuracy.

The main limitation of our study is the higher rate of lost to follow-up in the threaded group (n = 5 implants, 26%) compared to the microthreaded group (n = 1, 0.05%). Although overall lost to follow-up was 14% and within the acceptable range of ≤ 20% drop out, but the missing pattern was not at random [[52, 53] Therefore, it should be considered in the interpretation of outcomes and cannot be concluded with certainty.

## Conclusion

Within the limitation of this study, any superiority of the microthread design compared to threaded one could not be demonstrated in terms of implant success and marginal bone preservation around immediately placed implants over 5 years of loading.

## Data Availability

The datasets used and/or analyzed during the current study are available from the corresponding author on reasonable request.

## Abbreviations

MBL: Marginal bone loss
PI: Plaque index
BOP: Bleeding on probing
SUP: Suppuration
